# The Effect of (E)-1-(4’-aminophenyl)-3-phenylprop-2-en-1-one on* MicroRNA-18a*, *Dicer1*, and *MMP-9* Expressions against DMBA-Induced Breast Cancer

**DOI:** 10.31557/APJCP.2020.21.5.1213

**Published:** 2020-05

**Authors:** Ida Ayu Ika Wahyuniari, I Gusti Kamasan Nyoman Arijana, Ni Putu Sriwidyani, Hery Suwito, Sitarina Widyarini, Muhammad Ghufron, Mustofa Mustofa, Sofia Mubarika Haryana

**Affiliations:** 1 *Department of Histology, Faculty of Medicine, Udayana University, Bali, Indonesia. *; 2 *Department of Anatomical Pathology, Faculty of Medicine, Udayana University, Bali, Indonesia. *; 3 *Department of Chemistry, Faculty of Science and Technology, Universitas Airlangga, Surabaya, Indonesia. *; 4 *Department of Pathology, Faculty of Veterinary Medicine, Universitas Gadjah Mada, Yogyakarta, Indonesia. *; 5 *Department of Histology and Cell Biology,Universitas Gadjah Mada, Yogyakarta, Indonesia. *; 6 *Department of Pharmacology and Therapy, Faculty of Medicine, Universitas Gadjah Mada, Yogyakarta, Indonesia.*

**Keywords:** Chalcone, miR, 18a, Dicer, MMP-9, mammary cancer

## Abstract

**Background:**

Most of breast cancer patients are estrogen receptor alpha-positive and have high resistance and side effect of chemotherapeutic drug. Therefore, discovering an effective anticancer agent is needed. This research explored the effect of (E)-1-(4’-aminophenyl)-3-phenylprop-2-en-1-one (APE) on *miR-18a*, *Dicer1*, and *MMP-9 *expressions.

**Methods:**

Twenty four female Sprague-Dawley rats were invetigated in this study. The rats were divided into 6 groups of 4. G1 was considered as normal rat. G2, G3, T1, T2, and T3 were given DMBA 20 mg/kgBW twice a week for 5 weeks to induce mammary cancer. After being affiliated with cancer, G2 was given vehicle and G3 was treated with tamoxifen. T1, T2, and T3 were treated with APE intraperitoneally everyday for 21 days at doses of 5, 15, and 45 mg/kgBW/day, respectively. Blood plasma was collected to measure *miR-18a* expression using qRT-PCR. Mammary tissues were also collected to determine* Dicer1* and *MMP-9* expressions by using immunohistochemistry.

**Results:**

The results showed significant down-regulation of miR-18a relative expression and up-regulation of *Dicer1* expression in G3 and T1 compared to G2 (P<0.05). *MMP-9* expression has significant decrease in T1 compared to G2 (P<0.05).

**Conclusion:**

APE can decrease *miR-18a* and *MMP-9 *expressions and increase* Dicer1* expression in rat mammary cancer. Therefore, this compound could be a candidate of novel anticancer.

## Introduction

Millions of women are diagnosed with breast cancer every year around the world. It is the most frequent malignancy in women (Akram et al., 2017; Zehni et al., 2019). Most of all breast cancer patients (70%) are estrogen receptor alpha (ERα)-positive (Hayes and Lewis-Wambi, 2015; Zehni et al., 2019). Chemotherapeutic drugs, such as tamoxifen, have many undesirable side effects and their efficacy against carcinoma is still unsatisfactory. Nearly, 40-50% of patients with estrogen receptor-positive breast cancer develop resistance towards tamoxifen. In addition, the five-year survival rate after tamoxifen-resistance is less than 20%. Tamoxifen-resistance is often presented in the form of tumor recurrence and/or distant metastasis in most cases (Tanic et al., 2012; Zhao and Ramaswamy, 2014; Rondon-Lagos et al., 2016; Sotgia et al., 2017). The recurrence rate of ERα-positive breast cancer is 90% in Luminal B and 30% in Luminal A (Sas et al., 2012). Therefore, much more attention on the discovery of effective anticancer agents is needed.

The medicinal chemists of natural product have been explored for drug discovery of potential anti-cancer agents, such as flavonoids given their antioxidant and cytotoxic properties (Pande et al., 2017). Flavonoid regulates *miRNA* expressions through epigenetic modification, transcription factors modulation, and miRNA maturation process (Srivastava et al., 2015). Chalcones belong to flavonoid family that has been shown to exert many properties for human diseases, including anticancer (Jin et al., 2013). 

A series of new chalcone derivatives had been successfully synthesized to discover new anticancer, such as (e)-1-(4’-aminophenyl)-3-phenylprop-2-en-1-one (APE) (Suwito et al., 2015). Previously, the potential cytotoxicity of this compound against breast cancer cell line had been proven reducing tumor growth and down-regulating miR-21 in rat mammary cancer (Wahyuniari et al., 2017). However, lack of study regarding mechanism of action of this compound in metastasis effect. 

Loss of *Dicer* expression is associated with the progression and metastasis of breast cancer (Khoshnaw et al., 2012). *Dicer* expression is significantly lower in triple-negative breast cancer (TNBC) versus estrogen receptor-positive (ER+) clinical specimen of primary tumor. Overall, TNBC has poor prognosis in comparison with ER+ breast cancer (Spoelstra et al., 2016). Dicer is a Ribonuclease III enzyme playing a crucial role in microRNAs (miRNA) maturation from pre-miRNAs molecules (Price and Chen, 2014). As we know, miRNA is an important epigenetic mechanism which acts as a negative gene regulator by binding to mRNA. Epigenetic is potential target in cancer treatment and prevention due to its modifiable nature (Basse and Arock, 2015). Furthermore, the activity of more than 60% of all protein-coding genes is predicted to be controlled by miRNAs in mammals (Catalanotto et al., 2016). Interestingly, Dicer1 is also regulated by miRNA. Hence, over-expression of *miRNAs* targeting *Dicer1* leads to global down-regulation of *miRNA* expression (Luo et al., 2013). 

One of the miRNAs that regulates Dicer is miR-18a through its affinity with the 3’ untranslated region (Luo et al., 2013; Chen et al., 2014). MiR-18a also regulates ERα (Howard and Yang, 2018); however, Dicer is more significant than ER as a prognostic factor (Khoshnaw et al., 2012). In breast cancer, the expression of miR-18a increases (Shidfar et al., 2016), but the expression of *Dicer1* decreases (Yan et al., 2012). A previous study showed that increased *miR-18a* expression targeting Dicer-1 in nasopharyngeal cancer led to 78% decreased *miRNA* expression, including miR-143 (Luo et al., 2013). Decrease in *miR-143* expression leads to increase of matrix *metalloproteinase-9 (MMP-9)* expression due to* MMP-9 *as target molecule of miR-143 (Abba et al., 2014). MMP-9 degrades protein in extracellular matrix and it is associated with tumor invasion, metastasis, and poor prognosis in breast cancer (Merdad et al., 2014; Yousef et al., 2014). In addition, the increase of *MMP-9* expression can be also due to insufficiency of PTEN (Chiang et al., 2016), which is also a binding target for miR-18a (Zhang et al., 2016). 

The current study aimed to investigate APE effect on miR-18a, Dicer1, and MMP-9 as molecular targets. Given that animal model may generate ERα-positive breast cancer on Sprague Dawley rat (Abba et al., 2016; Alvarado et al., 2017), we first determined the molecular mechanism by which APE inhibited invasiveness against 7,12-dimethylbenz(a)anthracene (DMBA)-induced breast cancer. 

## Materials and Methods

*Tested compound and animals*

We tested (e)-1-(4’-aminophenyl)-3-phenylprop-2-en-1-one (APE) in this experimental research. It was synthesized by (Suwito et al., 2015) at Department of Chemistry, Faculty of Science and Technology, Universitas Airlangga, Indonesia. This in vivo research was a randomized post-test only control group design using twenty four female Sprague-Dawley rats with the aged of 3-4 weeks were provided by Laboratorium Penelitian dan Pengujian Terpadu, Universitas Gadjah Mada (UGM). This study was approved by Medical and Health Research Ethics Committee Faculty of Medicine, UGM. The rates were caged individually in animal house of the Department of Pharmacology and Therapy, Faculty of Medicine, UGM. They were maintained on a 12 h light–dark cycle at 24°C. Rats were fed a standard diet. They were given ad libitum access to water. 

The rats were randomly divided into 6 groups of 4. Group 1 (G1) was treated with corn oil as control group. Other groups (T1, T2, T3, G2, G3) were given chemical carcinogen dimethylbenz(a)antracene (DMBA) 20 mg/kgBW (Sigma-Aldrich, St Louis) dissolved in corn oil twice a week for five weeks to induce mammary cancer (Meiyanto et al., 2007). Tested compound was dissolved with vehicle (saline: tween 80: DMSO = 8: 1: 1). Upon the appearance of mammary cancer, G2 (mammary cancer) was given vehicle and G3 (mammary cancer + tamoxifen) was treated with tamoxifen citrate (tamofen 10, Kalbe Farma, Indonesia) 6.6 mg/kgBW. All T (mammary cancer + APE) groups were treated with APE dissolved in vehicle intraperitoneally every day for 21 days at the doses of 5, 15, and 45 mg/kgBW, respectively (Wahyuniari et al., 2017).

*Analysis of qRT-PCR*

Qiagen miRNeasy plasma kit (Cat#217184) and miRNeasy plasma spike-in control (Cat#219610) were used to extract microRNA in accordance with the manufacturer’s protocol. Synthesis of cDNA was based on the protocol of Qiagen miscript II RT Kit (Cat#218160). The level of miRNAs was quantified by using MyGo Mini Real-time PCR (IT-IS Life Science, UK) and Qiagen miScript SYBR Green PCR Kit (Cat#218073). The primers used were rno-miR-18a specific primer, 5’-TAAGGTGCATCTAGTGCAGATAG-3’, and miScript universal primer (IDT, Singapore). The miR-18a levels of plasma used C. elegans miR-39 as internal control. They were normalized with the comparative Ct method relative to this exogenous miRNA. The fold change in expression of the target gene relative to the internal control gene was calculated using 2^-ΔΔCT^ method (Wang et al., 2015b; Vigneron et al., 2016). 

*Immunohistochemistry*

Twenty formalin fixed and paraffin-embedded blocks of rat mammary cancer were cut from G2, G3, T1, T2, and T3 (6 μm thick) and mounted on polylysine-coated slides. After deparaffinization in xylene, slides were rehydrated through graded series of alcohol and washed in phosphate buffer saline (PBS). The slides were steam for antigen retrieval and incubated in a protein block. Slides were incubated with polyclonal anti Dicer1 protein antibody (rabbit anti-rat Dicer1, Bioss, USA, Cat#bs-6697R) with dilution 1: 100 and anti MMP-9 protein antibody (rabbit anti-rat MMP-9, Bioss, USA, Cat#bs-0397R) with dilution 1: 200 based on manufacturer’s instructions. Each IHC run included appropriate positive and negative controls. Immunohistochemical expression of *Dicer1* and *MMP-9 *was assessed using light microscope in five visual fields selected as previously mentioned (Wehrhan et al., 2012) with 400x magnification. The number of cytoplasmic staining according to its intensity of mammary cancer cells were used image raster 3 software. Intensity of the cytoplasmic staining and its distribution were assessed using the semi-quantitative scoring system (H-SCORE) The formula was as follows: H-SCORE = ƩPi (i + 1)/100, where Pi was the percentage for each intensity of mammary cancer cell’s cytoplasmic staining (0-100%) and i was the staining intensity with a value of 0 – 3 that referred to lack of, weak, moderate, and strong staining, respectively (Germeyer et al., 2014).

*Statistical analysis*

To analyze the relative expression of *miR-18a*,* Dicer1*, and *MMP-9* expression, SPSS (version 17.0) was used and one way ANOVA was run. P<0.05 was set for statistical significance.

## Results

*miR-18a expression*

**Figure 1:**
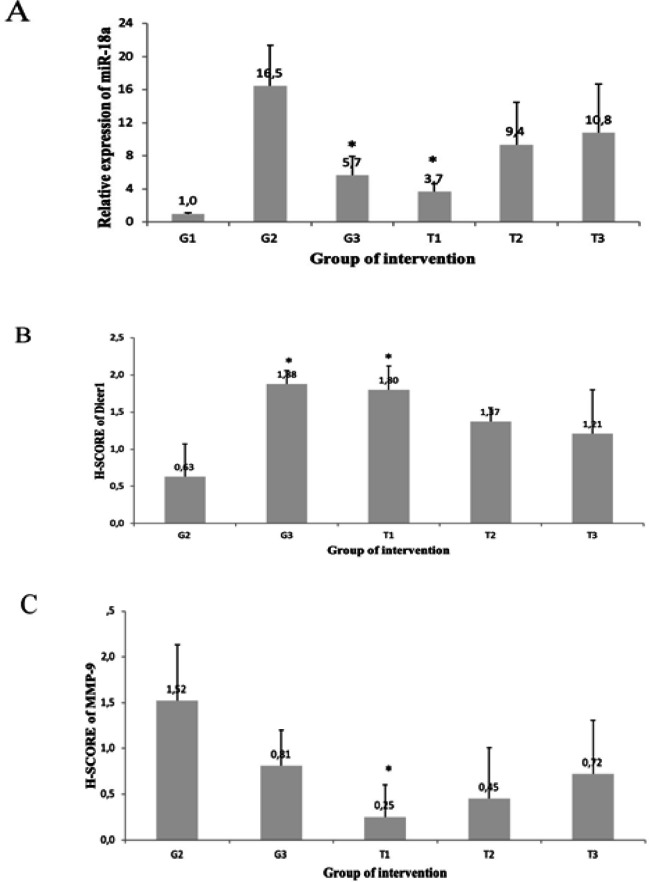
Effect of APE on miR-18a (A), Dicer1 (B), and MMP-9 (C) expression of rat mammary cancer. G1: normal rat, G2: mammary cancer rat, G3: mammary cancer + tamoxifen, T1: mammary cancer + APE 5 mg, T2: mammary cancer + APE 15 mg, T3: mammary cancer + APE 45 mg. *Significantly different from G2 (P<0.05).

The qRT-PCR examination revealed that *miR-18a* had lower expression in all T groups compared to G2 (G2=16.45 ± 4.89 ), G3=5.65 ± 2.29 ), T1=3.67 ± 0.95), T2=9.36 ± 5.09 ), and T3=10.80 ± 5.89) (Figure 1A). Statistical analysis showed significant lower result were only in groups G3 (p=0.011) and T1 (p=0.002) compared to G2. However, T2 and T3 was not significant different (p>0.05) compared to G2. 

*Dicer1 and MMP-9 expression*

**Figure 2:**
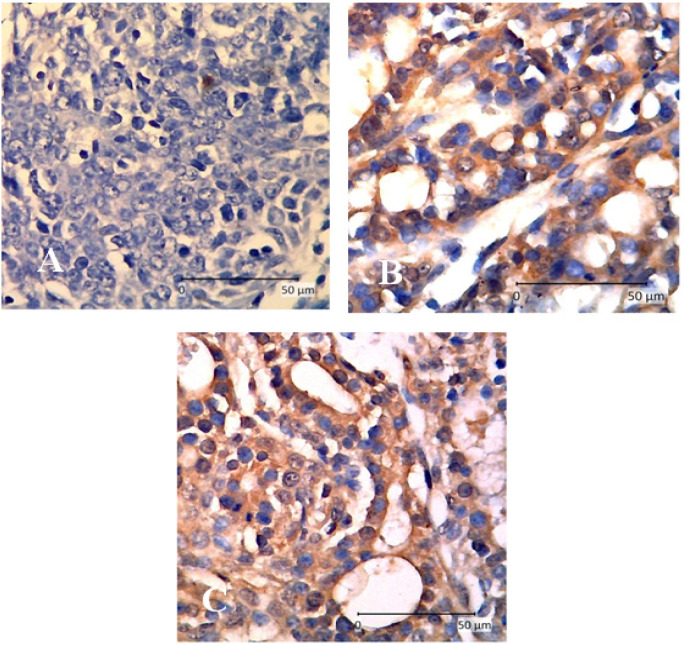
*Dicer1 *Expression in Rat Mammary Cancer with Immunohistochemistry (400x magnifications). A, mammary cancer rat (G2); B, mammary cancer + tamoxifen (G3); C, mammary cancer + APE (T1). Cytoplasmic staining was increased in treatment groups (B and C).

Figure 2 shows different cytoplasmic staining intensities of* Dicer1* expression in 400x magnification. We detected loss of Dicer1 in untreated mammary cancer (G2). H-SCORE of *Dicer1* expression was higher in treatment groups compared to untreated groups ( G2=0.63 ± 0.44, G3=1.88 ± 0.18 vs. T1=1.80 ± 0.32, T2=1.37 ± 0.19, and T3=1.21 ± 0.59). H-SCORE of *Dicer1* expression was significantly different in G3 and T1 (P=0.002) (Figure 1B). 

**Figure 3:**
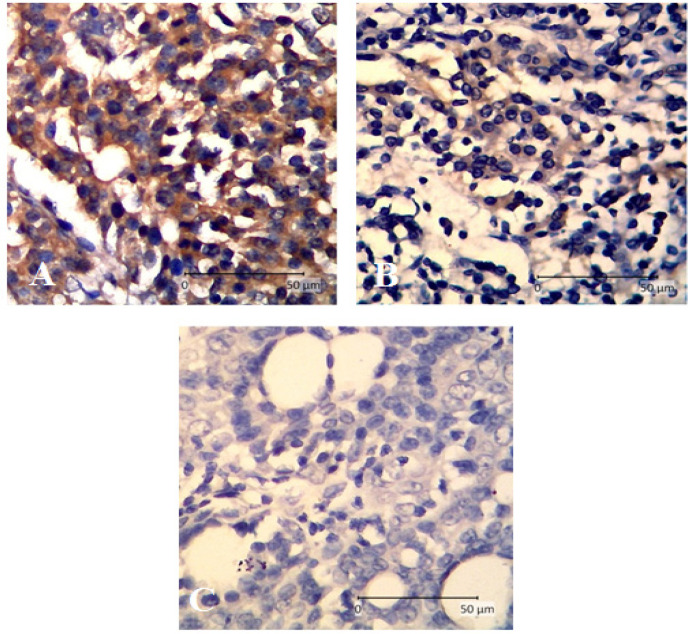
Immunohistochemistry of MMP-9 Expression in Rat Mammary Cancer (400x magnifications). A, mammary cancer (G2); B, mammary cancer + tamoxifen (G3); C, mammary cancer + APE (T1). Cytoplasmic staining was decreased in treatment groups (B and C).

According to Figure 1C, *MMP-9* expression decreased in all treatment groups compared to untreated ones ( G2=1.52 ± 0.61, G3= 0.81 ± 0.39 vs. T1= 0.25 ± 0.35, T2=0.45 ± 0.56, and T3=0.72 ± 0.59). The results was statistically significant only in Group T1 compared to G2 (P=0.030), while other groups were not significant (P>0.05). The expression of MMP-9 based on IHC in G2, G3, and T1 is shown in Figure 3, revealing prominent cytoplasmic staining of MMP-9 expression in group DMBA and vehicle. 

## Discussion

This study showed that APE at dose of 5 mg/kgBW could decrease *miR-18a* expression in this in vivo study. 

This compound belongs to chalcone derivatives as part of flavonoids family (Solomon and Lee, 2012). One study showed that flavonoids affected epigenetic mechanism including microRNA (Busch et al., 2015; Srivastava et al., 2015). Flavonoid regulates *miRNA *expression through modulation of transcription factors, epigenetic modification, and maturation of miRNA (Srivastava et al., 2015). This compound was also reported to down-regulate miR-21 (Wahyuniari et al., 2017). It was revealed that chrysin also down-regulated *miR-221*, *miR-21*, and *miR-18a *expressions in gastric cancer (Mohammadian et al., 2016)

The expression of *miR-18a* in this study was significantly higher in untreated rat mammary cancer (G2). Some studies showed similar results for breast cancer (Kodahl et al., 2014; Shidfar et al., 2016) and other cancers, such as nasopharyngeal cancer (Luo et al., 2013), gastric cancer (Tsujiura et al., 2015), esophageal cancer (Hirajima et al., 2013), pancreatic cancer (Morimura et al., 2011), colorectal cancer (Zhang et al., 2013; Yau et al., 2014), and hepatic cancer (Li et al., 2012). MicroRNA-18a is highly stable in blood; therefore, it is suitable as biomarker for non-invasive monitoring of tumor dynamics (Komatsu et al., 2014; Jin et al., 2015). MicroRNA can be a specific target for new anticancer agents due to its regulation on multiple target of mRNAs (Guo et al., 2013). 

Dicer1 mRNA is regulated by miR-18a through its affinity with the 3’ untranslated region of Dicer1 (Luo et al., 2013; Chen et al., 2014). In breast cancer, the expression of *miR-18a* increases (Shidfar et al., 2016) but the expression of* Dicer1* decreases (Yan et al., 2012). In this study, it was found that APE at the dose of 5 mg/kgBW significantly up-regulated *Dicer1* expression in T1. So, that is appropriate result that the decrease of *miR-18a* expression is followed by the up regulation of Dicer1 in Group T1 due to APE administration. According to a previous study, *Dicer* expression was positively correlated with disease free interval in 5 years. It meant that those patients who had high *Dicer *expression were less likely to have recurrence in 5 years compared to low *Dicer* expression patients. Dicer was thought to be more significant as prognostic factor than estrogen receptor in breast cancer patients (Khoshnaw et al., 2012). 

In this study, we found that Dicer1 had the lowest expression in rat mammary cancer without treatment (G2). Another study showed a gradual decrease of Dicer during progression of breast cancer, which was the strongest Dicer expression found in normal breast epithelial cells and the weakest in metastatic cells (Khoshnaw et al., 2012). Given that Dicer plays a crucial role in final maturation of miRNA (Price and Chen, 2014), Dicer dysregulation can lead to global disruption of *miRNA* expressions. Interestingly, Dicer1 is also regulated by miRNA. One study showed that up-regulation of miR-18a suppressed *Dicer* expression, causing global down-regulation of *miRNA* expression (78%), such as miR-143 (Luo et al., 2013). One of miR-143 target molecule is MMP-9 that is associated with tumor invasion, metastasis, and poor prognosis of breast cancer via protein degradation in extracellular matrix (Merdad et al., 2014; Yousef et al., 2014). 

In this study, anti-invasive control by APE was confirmed by the measurement of* MMP-9* expression. Down-regulation of MMP-9 was detected in groups treated with APE in 3 variation of dosage and tamoxifen, but significant only in group APE with dose 5 mg/kgBW. In addition, MMP-9 down-regulation may be also due to PTEN up-regulation which is also a binding target for miR-18a. We found APE capability to inhibit cancer progression. Other chalcone derivatives showed the same manner in metastasis inhibition. Synthetic chalcone, E-2-(40-methoxybenzylidene)-1-benzosuberone decreased secretion of MMP-9 (Pilatova et al., 2010). However, *MMP-2* expression was also decreased by novel anthraquinone based chalcone analogue (Kolundzija et al., 2014) and 2, 2-dimethylbenzopyran (Wang et al., 2015a).

The best response of APE was found in lower dosage. High dose of flavonoids may be toxic given their prooxidant activity and pro-inflammatory effects (Galati and O’Brien, 2004; Bouayed and Bohn, 2010; Corcoran et al., 2012). Further studies are suggested to find the optimal dosage of APE considering better efficacy of its lower dosage.

In conclusion, this study suggested that (E)-1-(4’-aminophenyl)-3-phenylprop-2-en-1-one (APE) may decrease miR-18a and increase Dicer1. This compound could inhibit invasiveness of breast cancer by decreasing MMP-9 expression. These results are substantial for the future development of this compound to control metastasis that often occurs in resistance and recurrence of breast cancer . 
